# Exposure to an insecticide formulation alters chemosensory orientation, but not floral scent detection, in buff-tailed bumblebees (*Bombus terrestris*)

**DOI:** 10.1038/s41598-024-65388-4

**Published:** 2024-06-25

**Authors:** Zsolt Kárpáti, Magdolna Olívia Szelényi, Zoltán Tóth

**Affiliations:** 1 Department of Chemical Ecology, Plant Protection Institute, HUN-REN Centre for Agricultural Research, Budapest, Hungary; 2 Department of Zoology, Plant Protection Institute, HUN-REN Centre for Agricultural Research, Budapest, Hungary; 3 National Laboratory for Health Security, Plant Protection Institute, HUN-REN Centre for Agricultural Research, Budapest, Hungary

**Keywords:** Foraging behaviour, Floral volatiles, Pesticide exposure, Wind-tunnel test, Sub-lethal effects, Mospilan, Chemical ecology, Animal behaviour, Entomology

## Abstract

Although pesticide-free techniques have been developed in agriculture, pesticides are still routinely used against weeds, pests, and pathogens worldwide. These agrochemicals pollute the environment and can negatively impact human health, biodiversity and ecosystem services. Acetamiprid, an approved neonicotinoid pesticide in the EU, may exert sub-lethal effects on pollinators and other organisms. However, our knowledge on the scope and severity of such effects is still incomplete. Our experiments focused on the effects of the insecticide formulation Mospilan (active ingredient: 20% acetamiprid) on the peripheral olfactory detection of a synthetic floral blend and foraging behaviour of buff-tailed bumblebee (*Bombus terrestris*) workers. We found that the applied treatment did not affect the antennal detection of the floral blend; however, it induced alterations in their foraging behaviour. Pesticide-treated individuals started foraging later, and the probability of finding the floral blend was lower than that of the control bumblebees. However, exposed bumblebees found the scent source faster than the controls. These results suggest that acetamiprid-containing Mospilan may disrupt the activity and orientation of foraging bumblebees. We hypothesize that the observed effects of pesticide exposure on foraging behaviour could be mediated through neurophysiological and endocrine mechanisms. We propose that future investigations should clarify whether such sub-lethal effects can affect pollinators’ population dynamics and their ecosystem services.

## Introduction

Pesticides are used to protect seeds and crops from weeds, herbivore pests and pathogens in agricultural production. In 2021, the total amount of active ingredients of pesticides used in agriculture reached 3.54 million tonnes worldwide, double the amount utilised in 1990^1^. However, due to processes such as (surface) runoff and drift, the vast majority of administered agrochemicals reach organisms other than their targets^2,3^, and pesticides thus often act as environmental pollutants, threatening human health^4^, ecosystem services^5^ and biodiversity^6^. Intensive agriculture inevitably relies on pesticide use to achieve global food security. Still, the continuous monitoring of their environmental impacts is essential to ensure that the application does not jeopardize the viability and functioning of affected (agro)ecosystems^7^.

Acetamiprid is an *N*-cyanoamidine neonicotinoid, which has widely been used as an insecticide in increasing quantity in both small gardens and large cultivations^8^ after the regulation change in the EU banned the more potent active ingredients of this group of compounds^9,10^. This substance is reported to be non-toxic to pollinators due to its rapid metabolisation^11^, yet recent investigations suggest that in high doses, it can induce various sub-lethal effects and thus pose a threat to pollinators^12,13^, natural predators of pests^14,15^ and other non-target organisms including vertebrates^16^. Previous investigations indicate that the field-relevant concentrations of acetamiprid can vary widely with specific environmental conditions and locations^17,18^, and even the concentrations of residues in flowers following application may range within several orders of magnitude (from 0.1 to 3360 ppb^19–21^), probably depending on the mode of application and crop type. Due to the apparent uncertainty associated with the exposure levels to this substance (including the exposure of humans to this neonicotinoid^22^), the sub-lethal effects of acetamiprid applied in agricultural production are likely to remain in the focus of ecotoxicology, conservation biology and health science research in the coming years.

Experimental results suggest that neonicotinoids may exert sub-lethal effects on insects by impairing olfactory detection and processing, thus subsequently affecting individual foraging behaviour. For instance, Andrione et al.^23^ found that acute imidacloprid treatment can disrupt honeybees’ odour coding and discrimination ability by reducing odour-induced calcium response in the antennal lobes. Antennal selectivity to common floral volatiles is also impacted by larval exposure to thiacloprid in this species^24^. Furthermore, clothianidin-exposed individuals show reduced antennal sensitivity in two bee species (*Osmia bicornis* and *Bombus terrestris*), with associated detrimental changes in foraging behaviour including a reduced number of flowers visited per flight and increased searching time in *O. bicornis*^25^. Similarly, exposure to imidacloprid reduces the activity of olfactory neurons in *Drosophila melanogaster*, and changes individuals’ relative preference for odour sources^26^. In *B. terrestris*, the same neonicotinoid negatively affects the motivation to initiate foraging bouts and reduces the amount of nectar collected^27^. However, different neonicotinoids can differentially influence bumblebee behaviour^28^, so it is unclear whether acetamipridinduced reductions in foraging performance can be attributed to impairments in peripheral olfactory detection, failure of information processing in the central nervous system, or linked to an overall activity-related deficiency (as suggested by^29^).

Bumblebees are known to provide essential pollination service in natural and agricultural ecosystems of temperate regions^30^, with implications for biodiversity and quality food production^31,32^. The foraging ranges of some *Bombus* species, such as *B. terrestris*, often include long-distance bouts, by which bumblebees can buffer against the adverse effects of heterogeneity amongst foraging patches and flowering crops^33^. If patch quality varies, chemosensory dysfunction in these species may substantially compromise foraging success, and due to the small colonies and annual life cycle characteristic of most bumblebees, the associated sub-lethal effects may have severe fitness consequences^34^.

In this study, we investigated whether a 3-week-long exposure to the insecticide formulation Mospilan (active ingredient: 20% acetamiprid) can disrupt olfactory detection and affect foraging decisions in *B*. *terrestris* workers by conducting choice tests on control and exposed individuals and subsequent electrophysiological recordings of their antennal response. As a floral scent source, we used the synthetic odour blend of the white mustard (*Sinapis alba*), an annual plant of Mediterranean origin belonging to the family *Brassicaceae*. This species is in part pollinated by bumblebees, cultivated worldwide and has a significant agronomic value due to its wide range of applications (see^35^). Blend composition and the ratios of the components were determined following Saunier et al.^36^. We predicted that the applied pesticide treatment might exert adverse sub-lethal effects by hindering exposed individuals (1) from detecting the volatile blend by reducing antennal sensitivity, i.e. low antennal response, (2) from orienting toward the blend source, i.e. decreased success or increased latency of locating the scent source, or (3) from initiating a foraging bout, i.e. increased latency to start foraging. We also assumed that these potential influences are not mutually exclusive and may manifest simultaneously.

## Results

### Foraging behaviour

**Figure 1:**
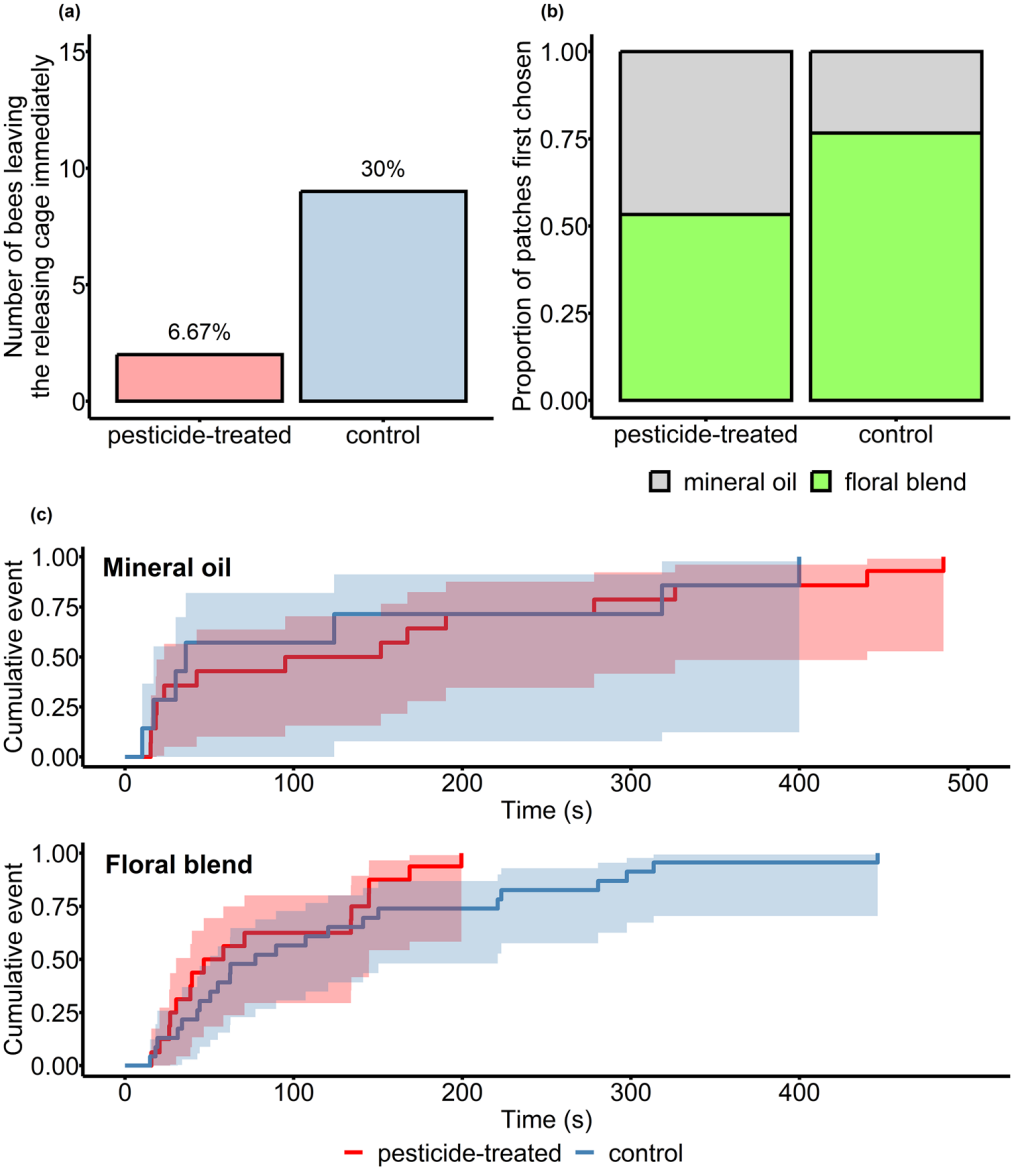
Behavioural responses of bumblebees to the present scent sources. (**a**) The number of focal individuals (blue: control; red: pesticide-treated) that left the releasing cage at the beginning of the behavioural trial without latency. Values on the top of the bars represent the number of individuals as a percentage of the total number of tested bees in each treatment group. (**b**) The proportion of focal individuals that approached the blend-containing (green) and the mineral oil-containing (grey) scent sources in the two treatment groups. (**c**) Kaplan–Meier curves for the cumulative incidences of the first scent source (upper panel: mineral oil; lower panel: floral blend) in the two treatment groups (blue: control; red: pesticide-treated). Curves are shown with 95% confidence intervals. For graphical presentation only, we used the *survfit* function of the ‘survival’ R package^72,73^ without including the random term.

We performed behavioural tests to investigate how control and exposed individuals differ in their orientation toward a mineral oil- and a synthetic blend-containing scent source in a wind tunnel. At the start of the trials, a higher percentage of bees left the releasing cage immediately (i.e., with no latency) in the control group than in the pesticide-treated group (*χ*^2^_1_ = 3.88, *P* = 0.049; Fig. 1a). Nevertheless, when individuals left the cage with latency, these latencies were similar in the two treatment groups (pesticide-treated [mean ± SD]: 13.55 ± 12.60 s; control: 13.50 ± 14.27 s). The starting time of trial did not affect this latency measure. The proportion of the blend-containing scent source as the first choice was significantly higher in the control than in the pesticide-treated group, indicating that untreated bumblebees located the blend first more successfully than pesticide-treated individuals (pesticide-treated: 16 out of 30 bumblebees, control: 23 out of 30 bumblebees; *χ*^2^_1_ = 3.87, *P* = 0.049; Fig. 1b). This difference also meant a significant deviation from the 50% expected by chance in the control (probability of success ± SE: 0.80 ± 0.08, *z*-ratio = 2.66, *P* = 0.016) but not in the pesticide-treated individuals (0.54 ± 0.11, *z*-ratio = 0.38, *P* = 0.707). The starting time of trial was also positively related to this measure (*χ*^2^_1_ = 4.76, *P* = 0.029), implying that the odds of choosing the blend-containing scent source increased over time (most likely due to increasing hunger) irrespective of the pesticide treatment. Pesticide-treated bees, however, approached the first scent source significantly sooner than control individuals did when it contained the synthetic blend (*χ*^2^_1_ = 4.13, *P* = 0.042; Fig. 1c). The latency to approach the first scent source when it contained only mineral oil did not differ between treatment groups. The starting time of trial had no significant effect on this measure at either scent source (Table 1).

**Table 1:** Test statistics and the significance of the explanatory variables from the fitted models. Significant predictors in the final models and their values are shown in bold; test statistics and *P*-values for the non-significant predictors were computed by including them one by one in the final models.

Response variable	Model type	Predictors	*χ*^2^	df	*P*-value
Antennal response (log(*x* + 0.1))	LMM with ARMA(*p*,*q*) correlation structure	Intercept	**451.03**	**1**	** < 0.001**
		Stimulus dose	**3308.80**	**4**	** < 0.001**
		Treatment	0.17	1	0.677
		Treatment × Stimulus dose	6.90	4	0.141
Latency to leave the releasing cage	Zero-inflated-Gamma GLMM—conditional component	Intercept	**184.90**	**1**	** < 0.001**
		Treatment	< 0.01	1	0.990
		Starting time of trial	1.38	1	0.240
	zero-inflation component	Intercept	**10.03**	**1**	**0.002**
		Treatment	**3.88**	**1**	**0.049**
		Starting time of trial	0.87	1	0.352
Type of the first-approached scent source	Binomial GLMM	Intercept	**4.48**	**1**	**0.034**
		Treatment	**3.87**	**1**	**0.049**
		Starting time of trial	**4.76**	**1**	**0.029**
Latency to reach the mineral oil first	Mixed-effects Cox model	Treatment	1.40	1	0.236
		Starting time of trial	0.44	1	0.504
Latency to reach the floral blend first	Mixed-effects Cox model	Treatment	**4.13**	**1**	**0.042**
		Starting time of trial	0.81	1	0.368

### Antennal detection

**Figure 2:**
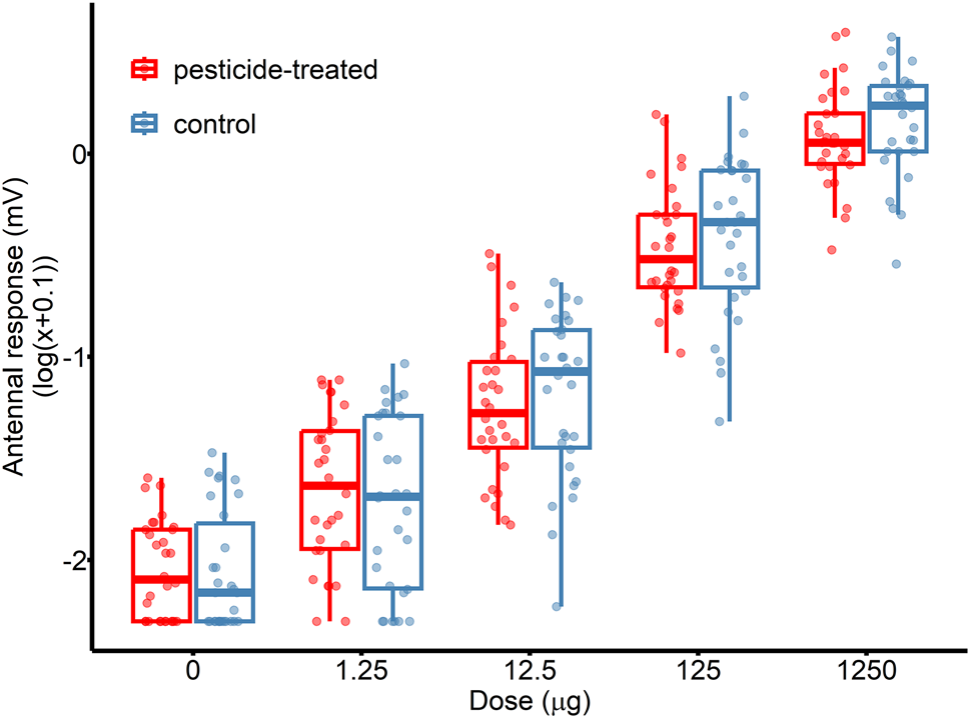
Electroantennographic dose responses of the focal individuals to the synthetic blend (*N* = 30 in both treatment groups). Boxplots show the median and interquartile range, whiskers indicate values within 1.5-fold of the interquartile range, and dots represent individual data points. The zero dose denotes the mineral oil control; we measured the EAG responses to this stimulus before and after the blend dose series and averaged the two measurements for each individual. Categories on the *x*-axis indicate the amount of volatile blend loaded into the stimulus cartridge.

We determined the impact of pesticide treatments on bumblebees’ floral volatile detection by examining the antennal electrophysiological responses of bumblebees’ to the synthetic floral blend after the wind tunnel test. The antennal responses of bumblebees were found to be dose-dependent (*χ*^2^_4_ = 3308.80, *P* < 0.001; Table 1; Fig. 2), with EAG amplitudes being the highest at the highest dose level. All doses significantly differed from the control (i.e., mineral oil) and from each other (including consecutive doses; Table 2). Pesticide treatment, however, had no significant effect on this measure either by itself or in interaction with the stimulus dose (both *P* ≥ 0.141).

**Table 2:** Result of the applied FDR post hoc tests for comparing the antennal responses between different stimulus doses. Estimates are given on the response scale.

Contrast	Estimate	SE	df	*t*-ratio	*P*-value
mineral oil – 1.25 µg blend	−0.06	0.01	4	−8.34	0.001
mineral oil – 12.5 µg blend	−0.17	0.02	4	−9.61	0.001
mineral oil – 125 µg blend	−0.52	0.05	4	−10.05	0.001
mineral oil – 1250 µg blend	−0.99	0.10	4	−10.17	0.001
1.25–12.5 µg blend	−0.11	0.01	4	−8.99	0.001
1.25–125 µg blend	−0.46	0.05	4	−9.99	0.001
1.25–1250 µg blend	−0.93	0.09	4	−10.11	0.001
12.5–125 µg blend	−0.35	0.04	4	−9.77	0.001
12.5–1250 µg blend	−0.82	0.08	4	−10.02	0.001
125–1250 µg blend	−0.47	0.05	4	−9.28	0.001

## Discussion

Chemosensory perception is crucial for all insects to locate food^37^. Thus, pesticide-induced changes in this process may have far-reaching consequences on food web relationships with plants and the provided ecosystem services in bumblebees^38,39^. We found no evidence that chronic exposure to an acetamiprid-containing formulation would influence antennal sensitivity to floral volatiles in buff-tailed bumblebee workers; however, we demonstrated several pesticide-induced alterations in their foraging behaviour. Specifically, we showed that a lower proportion of treated individuals started foraging immediately during the trials (6.7% vs. 30% of the tested bumblebees) and approached first the blend-containing scent source than the controls (53.3% vs. 76.7%). However, when the pesticide-treated bumblebees choose the floral blend first, they approach this source faster than the untreated animals (with the mean latency being 81.21 vs. 126.19 s). The observed behavioural changes imply that exposure to the Mospilan formulation affects both activity and orientation. Similar to other studies^27,40^, pesticide-exposed bumblebees were less motivated to initiate food searches but then reached the blend-containing scent source sooner; a faster initial flight speed in neonicotinoid-exposed bumblebees was also found by Kenna et al.^41^. On the other hand, treated individuals were not more attracted to the blend than to the odourless source (visual cues were present at both scent sources). These findings suggest that chronic exposure to this neonicotinoid can potentially reduce the resource-exploiting capacities of bumblebees by impairing their ability to locate high-quality patches signalled by concentrated floral scent.

We observed no adverse effect of the pesticide exposure on the olfactory detection of bumblebees despite previous findings showing strong support for reduced antennal sensitivity induced by other neonicotinoids^23–26^. However, the lack of effect is likely true only for the peripheral olfactory system. Neonicotinoids are known to act as potent agonists of the nicotinic acetylcholine receptors^42^, disturbing acetylcholine receptor signalling in the insect nervous system. This interference also occurs in the antennal lobes, which are the first odour-processing centres of the insect brain^23^. Moreover, experimental evidence indicates that Kenyon cells, major neuronal components of the mushroom bodies, may also become non-functional or unresponsive to excitatory synaptic input if neonicotinoids modulate the activity of nicotinic acetylcholine receptors^43^. Such effects, in turn, can lead to significant impairment of cognitive functions that are associated with these regions, including multisensory integration, spatial orientation and olfactory learning^44–46^. This mode of action would explain why pesticide-exposed bumblebees were not attracted to the blend source in our experiment but had the same sensitivity in the peripheral olfactory system.

As an alternative to the above mechanism or in parallel with it, the applied pesticide treatment may also alter foraging behaviour through other pathways. Neonicotinoids can act like endocrine-disrupting chemicals^47^, so low doses of these substances may modulate natural hormonal activities and interfere with gene expression^48^. Such disrupting effects of chronic acetamiprid exposure have been described in bumblebees related to locomotor activity: neurological symptoms such as abnormal stance, slow to no movements, and termination of normal daily activities like foraging^29^. This mechanism would explain, for instance, why treated bumblebees in this study were initially less motivated to start foraging than controls (as 4.5 times more bees started foraging without latency in the control group). As all observed behavioural alterations cannot be attributed to a single process, acetamiprid, like all other neonicotinoids, is likely to exert sub-lethal effects on different foraging parameters through several (neuro)physiological pathways, leading to varied and seemingly unrelated trait shifts.

Our study has two important limitations. On the one hand, we used colonies from the same batch, so our experimental design did not allow us to investigate inter-colony variation in antennal detection and scent source choice. In natural circumstances, colonies may differ profoundly in various behavioural parameters^29^. Therefore, the impact of these differences needs to be understood before we can generalise the observed effects of pesticide exposure on the entire species. On the other hand, we exposed bumblebees to 750–950 ppb concentrations of an acetamiprid-containing formulation for three weeks. Although this range represents a high but field-relevant dose following application^20,49^, continuous exposure to such concentrations is unlikely to occur under natural circumstances due to the expected degradation of the substance. Previous findings suggest that the post-application concentration of this neonicotinoid varies substantially depending on extrinsic factors, including the spraying technique and climatic conditions or crop type^19–21^. Besides, acetamiprid is known to degrade over time in soil and water due to, among others, microbial activity^50,51^, even though its concentration in aqueous solutions may remain unchanged for at least 5 days^52^. Not surprisingly, we also have very limited information on how the concentration of this substance changes over time in the nectar and pollen of flowering crops, which is the most relevant source of exposure to all pollinating insects. We propose that exposure levels similar to those applied in our study may nevertheless arise if bees feed from freshly sprayed flower patches for an extended period of time or if resources contaminated to this extent are stored in the colonies’ storage pots and get exploited frequently by colony members.

There is currently limited information on how acetamiprid affects olfactory perception and individual foraging behaviour in bumblebees^53^. Previously, acetamiprid was shown to increase antennal sensitivity; however, this investigation was conducted on honeybees following acute exposure^54^. In recent work, Tóth & Kovács^55^ found that acetamiprid treatments did not reduce syrup consumption but mediated how tested individuals exploited a second available food patch. Other studies focused only on this neonicotinoid’s effect on microcolonies’ overall syrup and pollen consumption^56–58^. The presented experimental findings are thus the first to imply that a field-relevant exposure to the Mospilan formulation can alter some aspects of foraging performance in bumblebees, likely by attenuating olfactory perception-related information processing in the central nervous system. Besides, pesticide-treated individuals also showed deviations from the locomotor activity of controls, probably due to an endocrine-disrupting effect of the pesticide. Similar works coupling individual- and colony-level sub-lethal effects could help to clarify whether chronic exposure to acetamiprid can jeopardise colony growth, population persistence or pollination service in this species. Such investigations are especially relevant as acetamiprid is expected to be used in large quantities^8,53^, leading to prolonged exposure of pollinators to this substance in agricultural areas^59^. Furthermore, neonicotinoid-substitute active ingredients, such as sulfoxaflor and flupyradifurone, should also deserve the attention of researchers because these substances may have similarly covert detrimental effects on pollinating insects^60^.

## Materials and methods

### Animal husbandry

We purchased 10 super-mini (containing > 40 workers) Biobest® hives of *B*. *terrestris* on 31^st^ August 2023 (Árpád Biokontroll 2003 Ltd). Upon our specific request, bees in the 10 hives originated from the same batch to avoid any substantial confounding colony-level effects on the behavioural measurements^29^. Colonies remained in their original brood box, with the top of the cardboard outer box left partly open for better ventilation. Bumblebees fed through an adjacent bottle containing sugar solution provided by the supplier (Biogluc®). In addition, we provisioned organic flower pollen (ApiLand SRL, Baia Mare, Romania) to the animals ad libitum. We randomly assigned the colonies to one of two treatment groups, control and pesticide-treated, and arranged the colonies randomly on the laboratory shelves concerning treatment. The ambient temperature was 24.4 ± 0.57 °C, and the relative humidity was 57.3 ± 2.77. Red light (Philips LED red bulbs; Philips International BV, Amsterdam, The Netherlands) has been used to illuminate the laboratory during the daytime (in a 13.5:10.5 h L:D).

### Preparation of floral volatiles

Based on Saunier et al.^36^, a synthetic floral volatile blend consisting of 10 synthetic compounds has been prepared. We combined the individual, synthetic, neat compounds in the following proportions: 2% of (*Z*)-3-hexen-1-ol (CAS: 928-96-1), 50% of benzaldehyde (CAS: 100-52-7), 8% of (*Z*)-3-hexenyl acetate (CAS: 3681-71-8), 1.4% of benzyl alcohol (CAS: 100-51-6), 0.2% of phenylacetaldehyde (CAS: 122-78-1), 3% of *β*-ocimene (CAS: 13877–91-3), 1.4% of acetophenone (CAS: 98-86-2), 2.8% of methyl salicylate (CAS: 119-36-8), 30% of anisaldehyde (CAS: 123-11-5) and 1.2% of *β*-caryophyllene (CAS: 87-44-5). For the electroantennogram (EAG) measurements, we dissolved the blend in mineral oil (CAS: 8042-47-5) and applied 10 µl of each dilution (0.125, 1.25, 12.5, and 125 µg/µl) to a filter paper disk (cotton liner, diameter: 12.7 mm; Carl Roth GmbH, Karlsruhe, Germany). Subsequently, the treated disk was inserted into a Pasteur pipette, serving as a stimulus cartridge. For the behavioural observations, we loaded 1–1 ml of the neat blend (as in^36^) and mineral oil as control stimulus in brown vial-wick dispensers^61^ and used these as two different types of scent source in the trials.

### Pesticide treatment and synthetic blend training

The pesticide exposure period took 21 days for each colony. We ensured the exact duration of exposure for all colonies by randomly appointing one control and one pesticide-treated colony to one of five exposure-starting days. Bumblebees from these pairs of colonies participated in the experimental tests on the same day (see below). We prepared a 0.4 g/L stock solution of Mospilan® 20 SG (20% acetamiprid content; Nippon Soda Co. Ltd., Japan; more details of the purchased formulation product can be found in the Supplementary Materials). As this formulation is water-soluble, we diluted 0.2 g of the granulate in 0.5 L of RO-filtered water (following the maximum recommended label rate). Then, we stored the stock solution at 4 ℃. On each exposure-starting day, we measured the volume of the Biogluc® syrup provided with the control and pesticide-treated colonies assigned to that given day. Then, we added the necessary volume of stock solution to create a syrup that contained a nominal concentration of 750 ppb acetamiprid for the pesticide-treated colony and added the same amount of filtered water (instead of the stock solution of the formulation) to the syrup for the control colony. When preparing the pesticide-treated Biogluc® syrup, we pipetted the appropriate amount of the stock solution (dependent on the volume of the syrup) into the syrup (also water-soluble) and homogenised the final solution. We administered the stock solution to the Biogluc® syrup only at the beginning of the 3-week-long exposure period. We performed a residual analysis using an Agilent (Santa Clara, California, USA) 5977C GC/MSD system to verify the concentration of acetamiprid in the prepared pesticide-containing syrup and to examine how it changed over time. For that, we sampled the syrup of each colony on the first and last day of the exposure period. This analysis confirmed that the administration of acetamiprid to the Biogluc® syrup was successful but also revealed that its concentration slightly increased over time, likely due to evaporation (mean (± s.d.) concentrations were 727.0 ± 115.31 ppb at the beginning and 927.12 ± 86.36 ppb at the end of the experiment). Thus, although the initial concentration of acetamiprid was within the range previously found in different crops after spraying^20,21^, representing a high but field-relevant dose, continuous exposure to such a concentration may rarely occur under natural circumstances. For more details on the residue analysis, see the Supplementary Materials.

We also pipetted 20 μl of the neat blend of synthetic floral volatiles into the Biogluc® syrup three times during the exposure period; thus, bumblebees could associate the scent with the nutritional reward.

### Behavioural observations

**Figure 3:**
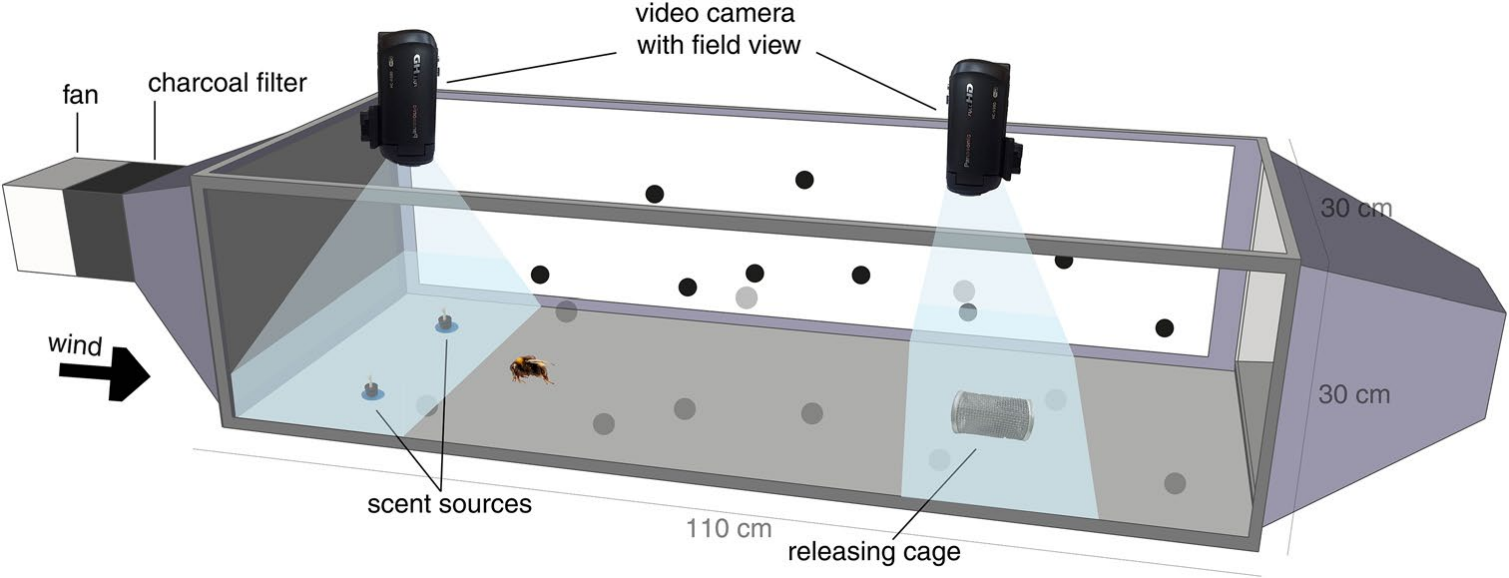
Schematic drawing of the wind tunnel. The releasing cage was 65 cm downwind from the scent sources (floral blend and mineral oil). Black filled circles were randomly placed on both inner sides of the wind tunnel providing visual cues for orientation. Video cameras recorded the scent sources and the releasing cage from above.

Behavioural trials took place in a wind tunnel (110 cm long × 30 cm wide × 30 cm high; Fig. 3), into which charcoal-filtered air at 22–23 °C and 59–65% RH was pushed through a fine-mesh screen at 0.1 m sec^−1^ airflow from the direction of scent sources toward the test animals. An exhaust expelled the wind tunnel air outside the building on the tunnel’s far end. The scent sources were two vial-wick dispensers sunk into the bottom of the wind tunnel (made of fibreboard); these dispensers were 8.5 cm from the sidewalls and 15 cm from each other. To facilitate orientation to the scent sources by visual cues, round disks (diameter: 2.4 cm) cut out from blue plastic sheets were attached below the black caps of the dispensers. The appointed pair of colonies was moved into the preparation room next to the wind tunnel one hour before the experiment. During a trial, we first placed the scent sources into their respective, randomly determined positions in the wind tunnel and then put a haphazardly selected individual from one of the two colonies (we determined the order in which the colonies were tested randomly) into an upside-down wire mesh cylinder (‘releasing cage’ henceforward; length: 5.5 cm, internal diameter [ID]: 3 cm) and left the focal individual in the cage for five minutes; the distance between the scent sources and the releasing cage was 65 cm. The room was lit only by a red LED light source during this period. After acclimatisation, the observer turned on the light (similar to daylight [5500–6500 K], provided by Osram Sylvania Luxline Plus light tubes), turned the releasing cage in a lateral position so the focal bee could leave the cage, and then left the room. During the following 10 min, we recorded the behaviour of the focal bee at the releasing cage and near the scent sources with two Panasonic HC-V380 video cameras from above. After 10 min, we turned off the light, so only a red light illuminated the premises again. We removed the focal individual from the wind tunnel in its releasing cage and put it aside for the EAG measurements. After each trial, the observer cleaned the releasing cage, the disks of the dispensers, and the wind tunnel using 70% ethanol and wiped with paper towels; the continuous airflow between trials ensured that no traces of the synthetic blend or alcohol were left in the tunnel.

### Antennal detection

To determine the impact of pesticide treatments on bumblebees’ floral volatile detection, we conducted electroantennography (EAG) recordings using the synthetic floral blend. All bumblebees were tested after their behavioural trial (i.e., within two hours on the same day). EAG recordings quantify the depolarisation amplitude of all responsive olfactory sensory neurons when exposed to a stimulus^62^. For the EAG recordings, the antenna of a *B. terrestris* worker was excised, half of the last (distal) segment was cut and both sides of the antenna were inserted into two glass electrodes (ID: 1.17 mm, Syntech, Kirchzarten, Germany), which were filled with Ringer solution^63^. The antennal signal underwent a pre-amplification of tenfold, was then converted into a digital signal using a DC amplifier interface (IDAC-2, Syntech), and subsequently recorded utilising GC-EAD software (GC-EAD 2014, version 1.2.5, Syntech). Antennae were stimulated for 0.5 s using a Stimulus Controller (CS-55, Syntech). The stimulation air (2 l/min) was directed into a consistently humidified and charcoal-filtered air stream (2 l/min). Mineral oil, the solvent of the floral volatiles, served as the control stimulus. Before and after each stimulus session, we measured the antennal responses of the focal individual to the mineral oil; within sessions, the four concentrations of the synthetic blend were applied in ascending order. We also counted the antennal segments after the EAG measurement to confirm that the tested individual was a worker.

### Video analysis

We analysed the video files with the event recorder BORIS 7.7.3^64^. To avoid observer bias, we renamed each video file to an identification number and analysed the recordings in a random order. We determined the following behavioural measures from the video recordings: time to leave the releasing cage after the observer turned the releasing cage in a lateral position and time to approach the first scent source. This latter was defined as an event when the focal individual gets so close to a scent source that its head crosses the edge of the blue disk attached to the dispenser or when the animal stops at the edge of the blue disk and starts cleaning its antennae (two instances).

### Statistical analysis

We tested 62 bumblebee workers, of which 31 were control and 31 were pesticide-treated individuals. We excluded two bees (one control and one pesticide-treated) from the subsequent analyses because they did not approach either scent source during the behavioural trial and had unusually low antennal responses even at the 12.5 µg dose in the EAG test. The EAG amplitudes of the mineral oil control stimulus measured before and after each stimulus session were averaged. In four instances, the antennal response of the animals to the 1.25 µg dose was lower than the averaged EAG amplitudes to the control; we replaced these values with the latter (i.e., allowed no lower response than to the control in the analysis). We obtained essentially identical results if we excluded these data points instead. All tests were performed in R 4.2.0.^65^. We used a linear mixed-effect model (LMM) with an autocorrelation-moving average correlation structure (*p* = 0, *q* = 2; ‘nlme’ R package^66^) to investigate how stimulus dose and pesticide treatment (both included as factors) affected the measured EAG amplitudes. The dependent variable was log-transformed (log(*x* + 0.1)) to improve its fit to normal distribution. We included ‘Individual identity’ nested within ‘Colony’ nested within ‘Day’ in this model as a random term. We applied a zero-inflated-Gamma generalised linear mixed model (GLMM) using Template Model Builder (‘glmmTMB’ R Package^67^) to examine how the starting time of trial (decimal time as a covariate) and pesticide treatment (as a factor) affected bumblebee’s latency to leave the releasing cage in the behavioural trial. By using a zero-inflated model, we were also able to take into account the frequent zero-valued observations. We fitted a GLMM with binomial error distribution to investigate how the type of the first-approached scent source (with the value of 1 for ‘blend’ and 0 for ‘mineral oil’) was affected by the starting time of trial and pesticide treatment. To check compliance with the assumptions of the (G)LMMs, we applied residual plot diagnoses (for GLMMs, we used the ‘DHARMa’ R package^68^). We used a mixed-effect Cox proportional hazards model to examine how the starting time of trial and the interaction between the type of the first-approached scent source and pesticide treatment affected the duration taken to approach the first scent source (‘coxme’ R package^69^). Due to the violation of the proportional hazard assumption for the predictor ‘scent source type’ when the model was fitted to the complete dataset, we ran the model separately on the ‘blend’ and ‘mineral oil’ datasets with starting time of trial and pesticide treatment as potential predictors. We included ‘Colony’ nested within ‘Day’ in the latter four mixed-effect models as a random term. To estimate the significance of potential predictors in the fitted models, we applied type III Wald *χ*^2^-tests using the Anova function of the ‘car’ R package^70^. Marginal means and post hoc comparisons with FDR adjustment were performed using the ‘emmeans’ R package^71^. All tests were two-tailed with α set to 0.05.

### Ethical note

No permits or approvals were required for the experiment on commercially available buff-tailed bumblebees in Hungary. After completing the experiment, we euthanised the animals by freezing them to avoid mixing with the natural population.

### Supplementary Information

Supplementary Information.

## Data Availability

Data files supporting the results are archived and available at Figshare (https://figshare.com/s/0d7f3e158c0ccdff8d0b).

## References

[1] FAO. Pesticides use and trade, 1990–2021. FAOSTAT Analytical Briefs Series No. 70., Rome; 10.4060/cc6958en (2023)

[2] Carriger JF, Rand GM, Gardinali PR, Perry WB, Tompkins MS, Fernandez AM (2006). Pesticides of potential ecological concern in sediment from South Florida canals: An ecological risk prioritization for aquatic arthropods. Soil Sedim. Contam..

[3] Pimentel D, Burgess M (2012). Small amounts of pesticides reaching target insects. Environ. Dev. Sustain..

[4] Smagghe, G. *et al.* Neonicotinoids and their substitutes in sustainable pest control. EASAC policy report 45. Schaefer Druck und Verlag GmbH (2023).

[5] Rumschlag SL (2020). Consistent effects of pesticides on community structure and ecosystem function in freshwater systems. Nat. Commun..

[6] Sánchez-Bayo F, Wyckhuys KA (2019). Worldwide decline of the entomofauna: A review of its drivers. Biol. Conserv..

[7] Vijver MG, Hunting ER, Nederstigt TA, Tamis WL, van den Brink PJ, van Bodegom PM (2017). Postregistration monitoring of pesticides is urgently required to protect ecosystems. Environ. Toxicol. Chem..

[8] Varga-Szilay Z, Pozsgai G (2023). Plant growers’ environmental consciousness may not be enough to mitigate pollinator declines: A questionnaire-based case study in Hungary. Pest Manag. Sci..

[9] EC. European Commission implementing regulation (EU) 2018/113. *OJEU* **20**, 7–10 (2018).

[10] EFSA (2016). Peer review of the pesticide risk assessment of the active substance acetamiprid. EFSA J..

[11] Decourtye, A. & Devillers, J. (2010). Ecotoxicity of neonicotinoid insecticides to bees in *Insect nicotinic acetylcholine receptors, advances in experimental medicine and biology* (ed Thany, S. H.), **683**, 85–95 (Springer, 2010). 10.1007/978-1-4419-6445-8_8 20737791

[12] Shi J (2020). Sublethal acetamiprid doses negatively affect the lifespans and foraging behaviors of honey bee (*Apis mellifera* L.) workers. Sci. Total Environ..

[13] Siviter H, Richman SK, Muth F (2021). Field-realistic neonicotinoid exposure has sub-lethal effects on non-Apis bees: A meta-analysis. Ecol. Lett..

[14] Řezáč M, Řezáčová V, Heneberg P (2019). Contact application of neonicotinoids suppresses the predation rate in different densities of prey and induces paralysis of common farmland spiders. Sci. Rep..

[15] Svoboda J, Pech P, Heneberg P (2023). Low concentrations of acetamiprid, deltamethrin, and sulfoxaflor, three commonly used insecticides, adversely affect ant queen survival and egg laying. Sci. Rep..

[16] Arican YE, Karaman EF, Özden S (2019). The sub-chronic effects of acetamiprid on the global DNA methylation levels in Sprague-Dawley rat brain and liver. Istanbul J. Pharm..

[17] Gupta S, Gajbhiye VT, Gupta RK (2008). Effect of light on the degradation of two neonicotinoids viz acetamiprid and thiacloprid in soil. Bull. Environ. Contam. Toxicol..

[18] Potts J, Jones DL, Macdonald A, Ma Q, Cross P (2022). Acetamiprid fate in a sandy loam with contrasting soil organic matter contents: A comparison of the degradation, sorption and leaching of commercial neonicotinoid formulations. Sci. Total Environ..

[19] Pohorecka K (2012). Residues of neonicotinoid insecticides in bee collected plant materials from oilseed rape crops and their effect on bee colonies. J. Apic. Sci..

[20] Stejskalová M, Konradyová V, Suchanová M, Kazda J (2018). Is pollinator visitation of *Helianthus annuus* (sunflower) influenced by cultivar or pesticide treatment?. Crop Prot..

[21] Capela N, Xu M, Simões S, Azevedo-Pereira HM, Peters J, Sousa JP (2022). Exposure and risk assessment of acetamiprid in honey bee colonies under a real exposure scenario in *Eucalyptus* sp. landscapes. Sci. Total Environ..

[22] Chen D (2020). Nationwide biomonitoring of neonicotinoid insecticides in breast milk and health risk assessment to nursing infants in the Chinese population. J. Agric. Food Chem..

[23] Andrione M, Vallortigara G, Antolini R, Haase A (2016). Neonicotinoid-induced impairment of odour coding in the honeybee. Sci. Rep..

[24] Ke L, Chen X, Dai P, Liu YJ (2023). Chronic larval exposure to thiacloprid impairs honeybee antennal selectivity, learning and memory performances. Front. Physiol..

[25] Straub F, Orih IJ, Kimmich J, Ayasse M (2021). Negative effects of the neonicotinoid clothianidin on foraging behavior and antennal sensitivity in two common pollinator species, *Osmia bicornis* and *Bombus terrestris*. Front. Ecol. Evol..

[26] Tatarko AR, Leonard AS, Mathew D (2023). A neonicotinoid pesticide alters *Drosophila* olfactory processing. Sci. Rep..

[27] Muth F, Leonard AS (2019). A neonicotinoid pesticide impairs foraging, but not learning, in free-flying bumblebees. Sci. Rep..

[28] Moffat C (2016). Neonicotinoids target distinct nicotinic acetylcholine receptors and neurons, leading to differential risks to bumblebees. Sci. Rep..

[29] Baines D, Wilton E, Pawluk A, de Gorter M, Chomistek N (2017). Neonicotinoids act like endocrine disrupting chemicals in newly-emerged bees and winter bees. Sci. Rep..

[30] Hutchinson LA (2021). Using ecological and field survey data to establish a national list of the wild bee pollinators of crops. Agric. Ecosyst. Environ..

[31] Johnson CA, Dutt P, Levine JM (2022). Competition for pollinators destabilizes plant coexistence. Nature.

[32] Bommarco R, Marini L, Vaissière BE (2012). Insect pollination enhances seed yield, quality, and market value in oilseed rape. Oecologia.

[33] Osborne JL (2008). Bumblebee flight distances in relation to the forage landscape. J. Anim. Ecol..

[34] Baron GL, Jansen VA, Brown MJ, Raine NE (2017). Pesticide reduces bumblebee colony initiation and increases probability of population extinction. Nat. Ecol. Evol..

[35] Mitrović PM (2020). White mustard (*Sinapis alba* L.) oil in biodiesel production: A review. Front. Plant Sci..

[36] Saunier A, Grof-Tisza P, Blande JD (2023). Effect of ozone exposure on the foraging behaviour of *Bombus terrestris*. Environ. Pollut..

[37] De Bruyne M, Baker TC (2008). Odor detection in insects: volatile codes. J. Chem. Ecol..

[38] Stanley DA, Garratt MP, Wickens JB, Wickens VJ, Potts SG, Raine NE (2015). Neonicotinoid pesticide exposure impairs crop pollination services provided by bumblebees. Nature.

[39] Stanley DA, Raine NE (2016). Chronic exposure to a neonicotinoid pesticide alters the interactions between bumblebees and wild plants. Funct. Ecol..

[40] Lämsä J, Kuusela E, Tuomi J, Juntunen S, Watts PC (2018). Low dose of neonicotinoid insecticide reduces foraging motivation of bumblebees. Proc. R. Soc. B.

[41] Kenna D, Cooley H, Pretelli I, Ramos Rodrigues A, Gill SD, Gill RJ (2019). Pesticide exposure affects flight dynamics and reduces flight endurance in bumblebees. Ecol. Evol..

[42] Brown LA, Ihara M, Buckingham SD, Matsuda K, Sattelle DB (2006). Neonicotinoid insecticides display partial and super agonist actions on native insect nicotinic acetylcholine receptors. J. Neurochem..

[43] Palmer MJ, Moffat C, Saranzewa N, Harvey J, Wright GA, Connolly CN (2013). Cholinergic pesticides cause mushroom body neuronal inactivation in honeybees. Nat. Commun..

[44] Stanley DA, Smith KE, Raine NE (2015). Bumblebee learning and memory is impaired by chronic exposure to a neonicotinoid pesticide. Sci. Rep..

[45] Stanley DA, Russell AL, Morrison SJ, Rogers C, Raine NE (2016). Investigating the impacts of field-realistic exposure to a neonicotinoid pesticide on bumblebee foraging, homing ability and colony growth. J. Appl. Ecol..

[46] Muth F, Francis JS, Leonard AS (2019). Modality-specific impairment of learning by a neonicotinoid pesticide. Biol. Lett..

[47] Christen V, Kunz PY, Fent K (2018). Endocrine disruption and chronic effects of plant protection products in bees: Can we better protect our pollinators?. Environ. Pollut..

[48] Vandenberg LN (2012). Hormones and endocrine-disrupting chemicals: low-dose effects and nonmonotonic dose responses. Endocr. Rev..

[49] Chandler AJ, Drummond FA, Collins JA, Lund J, Alnajjar G (2020). Exposure of the common Eastern bumble bee, *Bombus impatiens* (Cresson), to sub-lethal doses of acetamiprid and propiconazole in wild blueberry. J. Agric. Urban Entomol..

[50] Elango D (2023). Biodegradation of neonicotinoid insecticide acetamiprid by earthworm gut bacteria *Brucella intermedium* PDB13 and its ecotoxicity. Microbiol. Res..

[51] Guo L (2019). Biodegradation of the neonicotinoid insecticide acetamiprid by actinomycetes *Streptomyces canus* CGMCC 13662 and characterization of the novel nitrile hydratase involved. J. Agric. Food Chem..

[52] Ma X, Li H, Xiong J, Mehler WT, You J (2019). Developmental toxicity of a neonicotinoid insecticide, acetamiprid to zebrafish embryos. J. Agric. Food Chem..

[53] Varga-Szilay Z, Tóth Z (2022). Is acetamiprid really not that harmful to bumblebees (Apidae: Bombus spp.). Apidologie.

[54] El Hassani AK, Dacher M, Gary V, Lambin M, Gauthier M, Armengaud C (2008). Effects of sublethal doses of acetamiprid and thiamethoxam on the behavior of the honeybee (*Apis mellifera*). Arch. Environ. Contam. Toxicol..

[55] Tóth Z, Kovács Z (2024). Chronic acetamiprid exposure moderately affects the foraging behaviour of buff-tailed bumblebees (*Bombus terrestris*). Ethology.

[56] Camp AA, Batres MA, Williams WC, Koethe RW, Stoner KA, Lehmann DM (2020). Effects of the neonicotinoid acetamiprid in pollen on Bombus impatiens microcolony development. Environ. Toxicol. Chem..

[57] Camp AA, Williams WC, Eitzer BD, Koethe RW, Lehmann DM (2020). Effects of the neonicotinoid acetamiprid in syrup on *Bombus impatiens* (Hymenoptera: Apidae) microcolony development. PLoS ONE.

[58] Straub F (2023). Land-use-associated stressors interact to reduce bumblebee health at the individual and colony level. Proc. R. Soc. B.

[59] Zioga E, White B, Stout JC (2023). Honey bees and bumble bees may be exposed to pesticides differently when foraging on agricultural areas. Sci. Total Environ..

[60] Siviter H, Muth F (2020). Do novel insecticides pose a threat to beneficial insects ?. Proc. R. Soc. B.

[61] Molnár BP, Tóth Z, Kárpáti Z (2017). Synthetic blend of larval frass volatiles repel oviposition in the invasive box tree moth, *Cydalima perspectalis*. J. Pest Sci..

[62] Roelofs, W.L. Electroantennogram assays: Rapid and convenient screening procedures for pheromones in *Techniques in Pheromone Research* (eds Hummel, H.E. & Miller, T.A.) 131–159 (Springer, 1984)

[63] Ephrussi B, Beadle GW (1936). A technique of transplantation for *Drosophila*. Am. Nat..

[64] Friard O, Gamba M (2016). BORIS: A free, versatile open-source event-logging software for video/audio coding and live observations. Methods Ecol. Evol..

[65] R Core Team. R: A language and environment for statistical computing. R Foundation for Statistical Computing, Vienna, Austria. URL https://www.R-project.org/ (2022).

[66] Pinheiro, J., Bates, D., DebRoy, S., Sarkar, D. & R Core Team nlme: Linear and Nonlinear Mixed Effects Models. R package version 3.1-157. https://CRAN.R-project.org/package=nlme (2021).

[67] Brooks ME (2017). glmmTMB balances speed and flexibility among packages for zero-inflated generalized linear mixed modeling. R J..

[68] Hartig, F. DHARMa: Residual Diagnostics for Hierarchical (Multi-Level/Mixed) Regression Models. R package version 0.4.6. https://CRAN.R-project.org/package=DHARMa (2022).

[69] Therneau, T. M. Coxme: Mixed effects cox models. R Package Version 2.2–18.1. https://CRAN.R-project.org/package=coxme (2022a).

[70] Fox, J. & Weisberg, S. *An R companion to applied regression* (3^rd^ ed.) (Sage, 2019). https://socialsciences.mcmaster.ca/jfox/Books/Companion

[71] Lenth, R. Emmeans: Estimated marginal means, aka least-squares means. R package version 1.8.6. https://CRAN.R-project.org/package=emmeans (2023).

[72] Therneau, T. M. A Package for Survival Analysis in R. R package version 3.3–1. https://CRAN.R-project.org/package=survival (2022b).

[73] Therneau, T. M., & Grambsch, P. M. *Modeling survival Data: Extending the cox model.* (Springer, 2000).

