# Supplementary material for: Exposure to an insecticide formulation alters chemosensory orientation, but not floral scent detection, in buff-tailed bumblebees (Bombus terrestris)
Source: Sci Rep. 2024 Jun 25;14:14622. doi: 10.1038/s41598-024-65388-4 (PMC11199514; doi:10.1038/s41598-024-65388-4)
Supplement: Supplementary file 1 — Supplementary Information. [file 41598_2024_65388_MOESM1_ESM.docx]

**Supplementary Materials**

**Exposure to** **an insecticide formulation alters chemosensory orientation, but not floral scent detection, in buff-tailed bumblebees (*Bombus terrestris*)**

Zsolt Kárpáti^1^, Magdolna Olívia Szelényi^1,3^ & Zoltán Tóth^2*^

^1^Department of Chemical Ecology, Plant Protection Institute, HUN-REN Centre for Agricultural Research, Budapest, Hungary

^2^Department of Zoology, Plant Protection Institute, HUN-REN Centre for Agricultural Research, Budapest, Hungary

^3^National Laboratory for Health Security, Plant Protection Institute, HUN-REN Centre for Agricultural Research, Budapest, Hungary

**Formulation containing acetamiprid**

We purchased Mospilan® 20 SG in a 4 g package (manufacturer: Nippon Soda Co. Ltd., Japan; distributor: Sumi Agro Hungary Ltd., Hungary; packaging: CHEMARK Zrt., Hungary; production/batch/lot number: 2017310; production date: 01.01.2022; packaging date: 29.03.2022) from the Keszikert Budakeszi tree nursery and farm shop (25 Kert Str., H-2092 Budakeszi, Hungary) on 10^th^ February, 2023. We used this acetamiprid-containing formulation to prepare a 0.4 g/L stock solution for the experiment.

**Residue analysis**

To determine the concentration of acetamiprid at the beginning and at the end of the experiments in the Biogluc® syrup, we took samples from each colony and analysed by GC-MS in three technical replicates.

*Sample preparation*

Samples were mixed with dichloromethane in a 1:4 ratio, and mixed in laboratory thermoshakers overnight at 25°C. After mixing, the samples were centrifuged for 30 minutes at 13,300 rpm. The upper layer containing the sugar solution was removed by pipetting. To ensure that all of the upperlayer was removed, the samples were vortexed and centrifuged again. After that 100 µL was pipetted into a GC-vial containing a 200 µL volume insert for gaschromatograph coupled with mass-spektrometer (GC-MS) measurment; the remaining part was stored at -40°C.

*Gas-chromatography mass-spectrometry analysis*

Measurements were carried out on an Agilent (Santa Clara, California, USA) 5977C GC/MSD system. The injection port was set to 250 °C, the injection volume was 1 µl in splitless mode. Carrier gas of helium 6.0 was used at the column and the flow rate was 1 mL/min in constant linear velocity mode. The separation was performed on an Agilent J&W HP-5MS UI (30 m × 250 μm × 0.25 µm) nonpolar capillary column. The temperature program for separation started with a 2 min 70 °C hold, then increased by 20 °C/min to 300 °C and held for 5 min. As a post-run, the temperature was raised to 325 °C and held for 5 min before returning to initial conditions. For mass spectrometric detection, the source temperature was set to 230 °C while the quadrupole temperature was held at 150 °C. Positive electron ionization (EI+) was used with a standard electron energy level of 70 eV. The instrument was tuned using perfluorotributylamine according to the manufacturer’s instructions. First, authentic standards were injected in scan mode to develop a Selected Ion Monitoring (SIM) method for quantitative mass spectrometric detection and to confirm the compound by its mass spectrum utilizing the NIST 17 mass spectral database. For quantitative measurements, the MS was operated in SIM mode at a cycle time of 20 Hz. For the acetamiprid identification, the following ions were monitored: m/z 56, 126, 152, 166, 221. For quantitation, the 152 fragment was used. Agilent MassHunter Workstation CG/MS Data Aquisiton 10.2 was used to set the GC and MS parameters. For qualitative identification, validation and quantitative evaluation MassHunter Workstation Qualitative Analysis Navigator B.10.00 was used.

*Extraction method validation*

The validation of the analytical method was performed to determine the linearity of calibration, limit of detection (LOD), limit of quantification (LOQ) and evaluated the recovery rate of the sample preparation from the results of spiking experiments.

To determine recovery and matrix effects for our analytical assay, we performed pre-spike and post-spike experiments. In all experiments, five concentrations of acetamiprid (0.25, 0.5, 0.75, 1, and 1.25 ng/µl) were used. The recovery rate of acetamiprid was calculated by analysing three replicate spiked samples and comparing with matrix-matched calibration standards for each concentration. In the pre-spike experiment, Mospilan was added to the Biogluc® syrup at the four concentrations, followed by sample preparation. In the post-spike experiments sample preparation were applied on Biogluc® syrup without Mospilan. Mospilan were diluted in methanol for a stock solution. This stock was diluted in dichloromethane further, and added to the samples after sample preparation. Average recovery rate was 10.4 ±5.3 % (Fig. S1, Fig. S2).

The LOD and LOQ were determined based on the standard deviation of y-intercept of the regression line (s) and the slope of the calibration curve (S) as LOD = 3.3(s/S) and LOQ = 10(s/S) in accordance with ICH (International Council for Harmonisation) guidelines. The calibration curve for the quantification of acetamiprid exhibited a linear relationship with an value of 0.95 (Fig. S3). LOD was 0.11 ng/µl, while LOQ was 0.33 ng/µl.

We calculated the averages and standard deviations of the acetamiprid concentrations measured in each pesticide-treated colony from the three technical replicates (Table S1); the means and standard deviations for all colonies at the beginning and at the end of the experiment were calculated from these values.

Table S1. The averages and standard deviations of the acetamiprid concentrations measured in the five pesticide-treated colonies.

| Colony ID | Acetamiprid concentration | | | |
| --- | --- | --- | --- | --- |
|  | beginning of the experiment | | end of the experiment | |
|  | average (ng/µl) | SD | average (ng/µl) | SD |
| 1 | 0.577 | 0.238 | 0.983 | 0.229 |
| 2 | 0.799 | 0.034 | 1.025 | 0.050 |
| 3 | 0.875 | 0.063 | 0.915 | 0.077 |
| 4 | 0.761 | 0.038 | 0.935 | 0.038 |
| 5 | 0.622 | 0.142 | 0.778 | 0.114 |


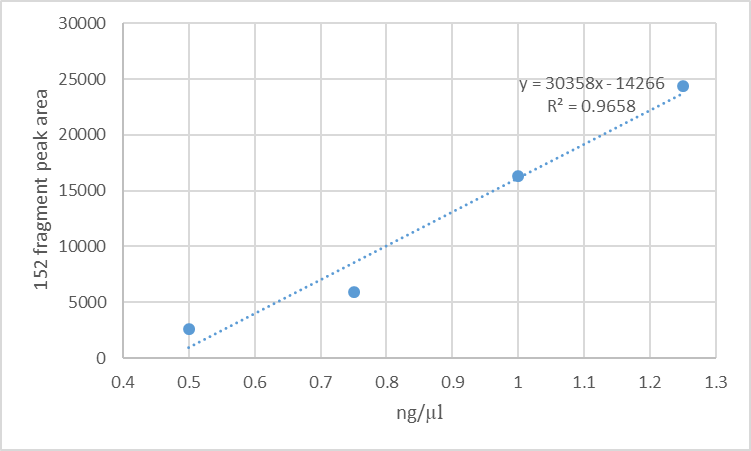


Figure S1. Dose curve of pre-spiking experiments.


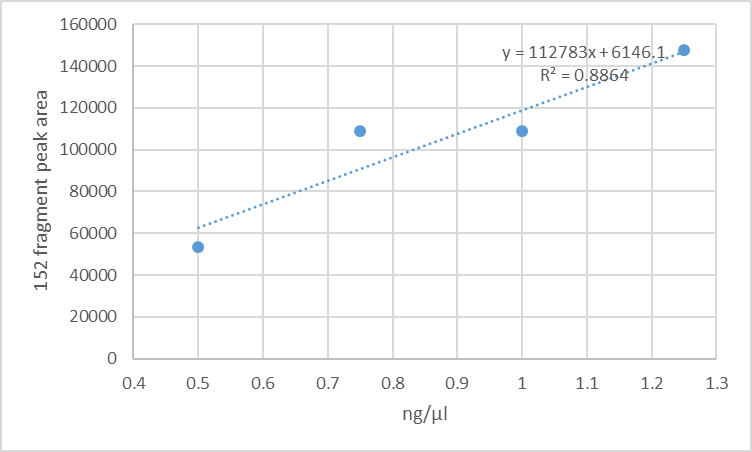


Figure S2. Dose curve of post-spiking experiments.


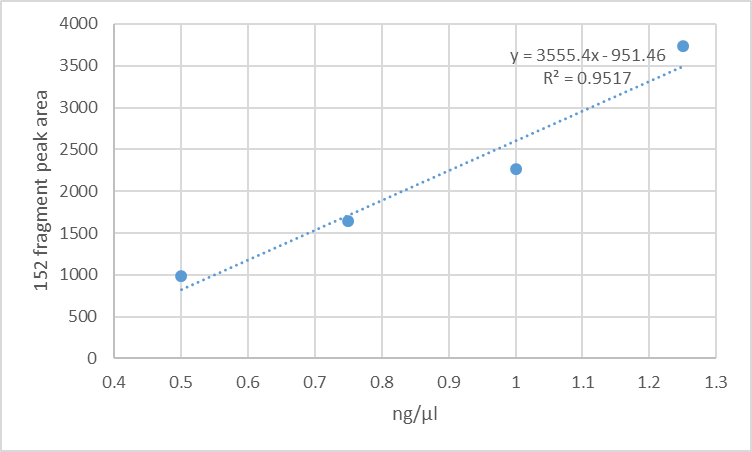


Figure S3. Dose curve of calibration.
